# Silicon Anodes for Next‐Generation Li‐Ion Batteries: Design, Metrics, and Practical Routes to 500 Wh kg^−1^ and 1000 Cycles

**DOI:** 10.1002/smsc.70347

**Published:** 2026-08-20

**Authors:** Mahesh Nepal, Tara P. Dhakal

**Affiliations:** ^1^ Center for Autonomous Solar Power (CASP) Binghamton University Binghamton New York USA; ^2^ Department of Electrical and Computer Engineering Binghamton University Binghamton New York USA; ^3^ Materials Science and Engineering Program Binghamton University Binghamton New York USA

**Keywords:** anode, Coulombic efficiency, energy density, materials science, nanoarchitectures for lithium‐ion batteries, nanotechnology, nanowires, power density, silicon, silicon anode, silicon microparticles, silicon nanoparticles, specific surface area, thin film silicon

## Abstract

Silicon (Si) anodes are key to enabling 500 Wh kg^−1^ lithium‐ion batteries for longer‐range electric vehicles, lightweight aerospace systems, and high‐energy consumer electronics. However, despite their high theoretical capacity (3579 mAh g^−1^), commercialization remains limited by severe volume expansion, unstable solid electrolyte interphase (SEI), low initial Coulombic efficiency (ICE), and insufficient long‐term Coulombic efficiency (CE). Here, we critically evaluate Si‐dominant anode architectures across three classes: nanoparticles and their secondary microspheres, micro‐silicon (µ‐Si), and structured Si, including nanowires and thin films supported on three‐dimensional scaffolds. Performance is benchmarked using practical capacity, ICE/CE, cycling stability, and volumetric metrics. We show that current Si technologies can realistically deliver ~400 Wh kg^−1^, with defined pathways toward ~500 Wh kg^−1^; however, achieving ≥1000 cycles remains a major challenge, primarily due to insufficient long‐term CE. Across architectures, high‐surface‐area electrodes, while beneficial for kinetics and strain accommodation, increase irreversible losses and limit cycle life. Strategies that reduce exposed surface area while maintaining transport and mechanical integrity, particularly through dense secondary particle design, offer a practical route toward scalable implementation. Progress toward commercial viability will require coordinated advances in interfacial passivation, contact‐preserving architectures, electrode densification, and manufacturing compatibility.

## Graphical abstract

Critical assessment of Si‐dominant anode architectures reveals that secondary‐particle designs offer the most practical route toward high‐energy Li‐ion batteries by bridging nanoscale performance with electrode‐level metrics, including volumetric capacity, cycling stability, and manufacturing compatibility.

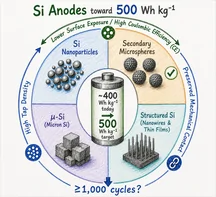

## 1 Introduction

Lithium‐ion batteries (LIBs), first commercially developed by Sony in 1991 [1], quickly became a leading energy storage technology across consumer electronics and, more recently, for electric transportation [2] and grid storage [3], owing to their favorable balance of energy and power density. As the market shifts from combustion engines to electric vehicles (EVs) and from fossil fuels to renewables, LIBs enable cleaner transportation and facilitate the grid integration of intermittent renewables, thereby supporting decarbonization. At the same time, emerging chemistries, such as sodium‐ion batteries [4], offer cost advantages for stationary storage and could challenge LIBs in that segment. For EVs, high‐energy‐density batteries are essential, as today's commercial LIBs (150–250 Wh kg^−1^) typically provide a driving range of approximately 200–300 miles per charge [5], short of the 500‐mile range target—a key factor in enhancing the appeal of EVs for long‐distance travel. To address this, the U.S. Department of Energy's Battery500 consortium aims to develop batteries with a specific energy of 500 Wh kg^−1^, which is nearly double the energy density of today's LIBs [6]. Achieving this target is difficult with conventional graphite anodes paired with lithium transition‐metal oxide cathodes (e.g., LiCoO_2_), whose capacities limit cell‐level energy. Advancing earth‐abundant, cost‐effective electrode materials with higher capacity offers a path to sustain and extend LIB dominance in the coming years.

Among anode materials, silicon (Si) is a promising candidate for next‐generation LIBs. It offers a very high theoretical specific capacity of 3579 mAh g^−1^ (more than nine times that of graphite), natural abundance as the second‐most‐abundant element in Earth's crust, and a higher lithiation potential than graphite (~0.4 V vs. Li/Li+ for Si compared to <0.2 for graphite), which lowers the risk of Li plating and enhances safety. While metallic lithium is often described as the “Holy Grail”, it faces significant practical challenges in terms of safety and cyclic stability, despite decades of research [7]. Because Li metal stores charge through an intrinsically nonuniform electroplating/stripping process, dendrites—hair‐like structures—can form and penetrate the separator, causing internal short circuits and severe safety risks. By contrast, Si anodes, which operate at a higher potential and thus mitigate Li‐plating while avoiding dendritic hazards, offer a practical route to higher cell‐level energy density. When paired with high‐capacity cathodes, they can push LIBs toward the 500 Wh kg^−1^ target for next‐generation EV cells.

## 2 Challenges With Silicon‐Based Anodes

**FIGURE 1:**
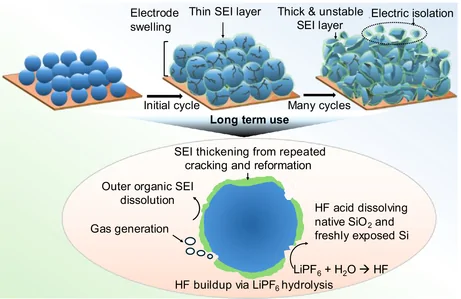
Challenges with Si anodes: severe volume expansion leading to structural degradation, affecting both cycle and calendar life.

Despite their great potential for enhancing LIB energy density, Si anodes face major barriers to practical application and commercialization. The most prominent is the extreme lithiation‐induced volume expansion, which can reach up to ~300% at full alloying to Li_15_Si_4_ (3.75 Li per Si atom). As lithium ions are inserted into compact Si, the interatomic distance within the Si lattice expands, putting significant mechanical stress on the Si particles. Repeated expansion/contraction during cycling fractures Si particles (pulverization), drives electrode swelling and cracking, and disrupts electronic pathways to the current collector (Figure 1), causing rapid capacity loss. In contrast, graphite forms LiC_6_ via intercalation of Li^+^ ions (atomic radius ~0.076 nm) between graphene layers (interlayer spacing ~0.335 nm), resulting in minimal volume expansion (<15%) [8]. The associated strain in Si also compromises the stability of the solid electrolyte interphase (SEI) layer: cyclic rupture exposes fresh Si, promoting continual SEI formation that consumes electrolyte and active lithium, decreases Coulombic efficiency (CE), and accelerates capacity fade. Progressive SEI thickening raises interfacial resistance, further degrading cell kinetics.

Another critical limitation of Si anodes is intrinsically poor charge transport. Si is a semiconductor with an intrinsic electrical conductivity of ~10^−5^ S cm^−1^, orders of magnitude less than that of graphite. Moreover, because Li storage in Si occurs via alloying rather than intercalation, Li must diffuse through the bulk Si matrix rather than along intercalating layers, making ion transport comparatively sluggish. During lithiation, the formation of Li_*x*_Si phase increases electronic conductivity and improves ionic transport [9]. However, during delithiation, the reverse occurs, and both electronic and ionic conductivities decline, hindering Li extraction and slowing electrode kinetics. The combination of low conductivity and sluggish ion diffusion limits rate capability and power output.

Furthermore, as Li‐ion cells age, additional degradation pathways emerge for Si anodes. A key driver is hydrofluoric acid (HF), generated through the hydrolysis of the commonly used electrolyte salt lithium hexafluorophosphate (LiPF_6_) in the presence of trace amounts of water in the cell [10]. While graphite anodes are relatively inert to HF [11], Si/SiO_2_ anodes are highly susceptible: HF readily etches native SiO_2_, producing soluble fluorosilicates (e.g., H_2_SiF_6_), gaseous products (e.g., SiF_4_), and water (SiO_2_ + 6HF → H_2_SiF_6_ + 2H_2_O; SiO_2_ + 4HF → SiF_4_ + 2H_2_O). The water generated further accelerates LiPF_6_ hydrolysis, amplifying HF production. While HF typically etches SiO_2_ in aqueous solutions, the nonaqueous electrolyte and extreme electrochemical conditions in batteries may allow HF and its by‐products to attack Si itself [11], likely through initial surface oxidation followed by dissolution. The resulting gaseous by‐products accumulate during storage, causing cell bulging and raising safety concerns. Compounding this, the SEI on Si anodes is highly unstable due to its dynamic nature, unlike the relatively stable SEI on graphite anodes enhanced by electrolyte additives. During lithiation, the SEI on Si becomes thinner and more inorganic, while during delithiation, it thickens and becomes more organic, attributed to the continuous precipitation and dissolution of SEI components [12]. Fluoroethylene carbonate (FEC) [13], a widely used additive, can enhance SEI stability and extend cycle life from tens to hundreds of cycles, but it does not fully prevent SEI instability or variations in SEI thickness [13]. Beyond cycle life, these issues strongly influence calendar life. While conventional graphite cells can exceed 15 years, Si‐containing cells, even with low Si content, typically degrade within 1–2  years [14]. For automotive use, a 10‐year calendar life and 1000‐cycle lifespan are key technical requirements [15].

## 3 Opportunities for Silicon in Next‐Generation Batteries

The development of LIBs began with lithium metal as an anode, first patented by Whittingham in 1977 while working at Exxon [16]. By the late 1980s, companies such as Moli Energy commercialized lithium metal batteries [17], but safety concerns—stemming from high reactivity, dendrite‐induced internal short circuits, and thermal runaway—prompted recalls and a shift toward safer alternatives. In 1991, Sony introduced LIBs with graphite anodes [1], which have since dominated the market due to their safety and stability. Despite lithium metal being the ideal anode due to its unmatched theoretical capacity (3860 mAh g^−1^), safety and practical challenges have constrained broad deployment to date. These limitations have shifted attention to Si, which offers the next‐highest capacity (3579 mAh g^−1^), and the potential to achieve energy densities above 500 Wh kg^−1^, making it a promising candidate for next‐generation Li‐ion batteries.

**FIGURE 2:**
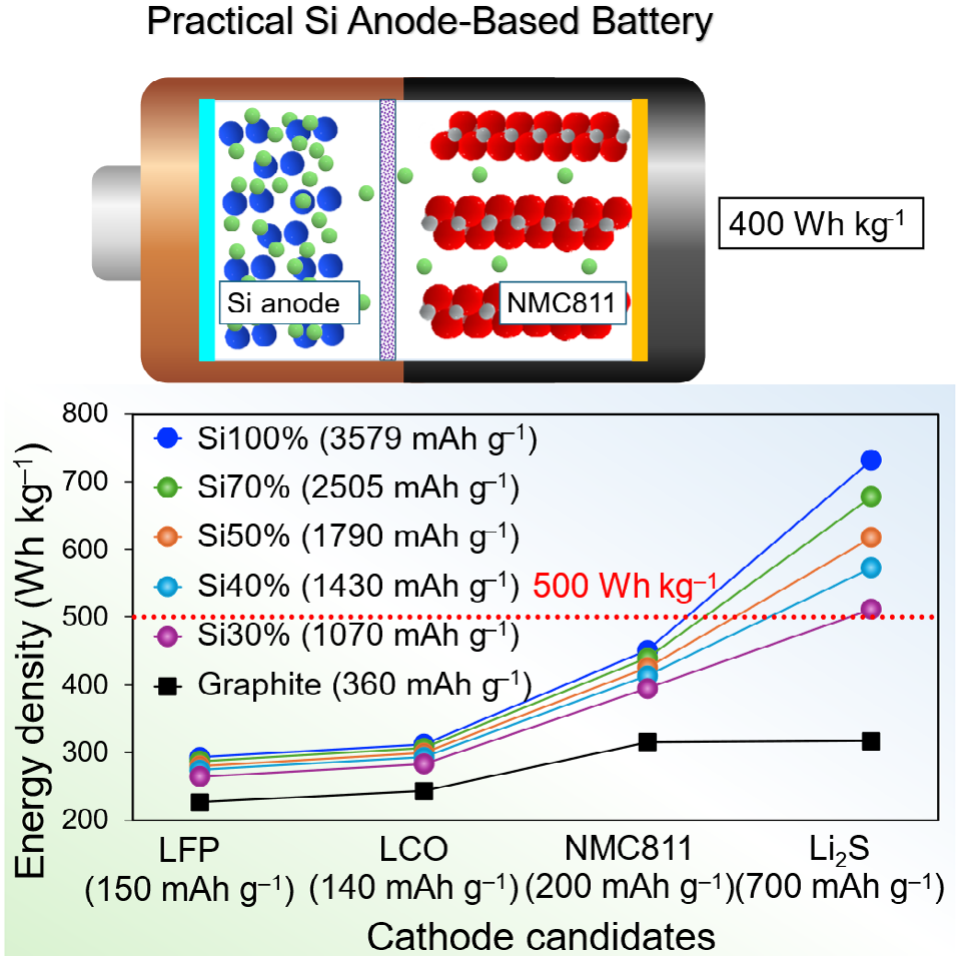
Practical energy density of Si‐based batteries: estimated practical energy density of Si‐anode batteries with commercial and sulfur cathodes, calculated considering practical constraints.

To quantify Si's advantages over graphite, we estimated cell‐level energy density (Wh kg^−1^) for full Li‐ion cells pairing Si anodes with commercial cathodes—LiFePO_4_ (LFP), LiCoO_2_ (LCO), and NMC811—as well as with a high‐capacity sulfur cathode (Li_2_S). While sulfur is not yet commercialized, its high theoretical capacity (1167 mAh g^−1^ for Li_2_S) makes it a promising candidate [18]. We consider a Si anode at various practical specific capacities corresponding to 30%, 40%, 50%, 70%, and 100% of the theoretical capacity (3579 mAh g^−1^) to reflect literature‐reported ranges; the results are shown in Figure 2. For reference, we performed the same analysis for a graphite anode (360 mAh g^−1^) using the same cathode pairings. Calculations proceed in two steps: first, determine material‐level full‐cell capacity (*C*_cell,mat_) including N/P ratio and AOH (anode overhang); second, translate to cell‐level energy density (E_cell_) by accounting for inactive components (current collectors, separators, electrolyte, tabs, packaging).

(1) $$C_{\text{full},\text{mat}}\left(\text{mAh}\;g^{-1}\right)\;=\;\frac{1}{\frac{N/P\;\times \;\left(1\;+\;\mathrm{AOH}\right)}{C_{a,\text{mat}}}\;+\;\frac{1}{C_{c,\text{mat}}}}$$

(2) $$E_{\mathrm{cell}\;}(\mathrm{Wh}\;{\mathrm{kg}}^{-1})=\;C_{\mathrm{full},\mathrm{mat}\;}\;\times V_{\text{nominal}}\;\times \eta$$

Here, N/P denotes the ratio of areal‐capacity of the negative electrode (anode) to that of the positive electrode (cathode). Because the cathode provides the initial lithium inventory, the anode is designed with excess areal capacity (N/P > 1) to reduce the risk of lithium plating during charging and provide a safety margin. An N/P ratio of  1.1 is used in this analysis. AOH is the deliberate extension of the anode beyond the cathode footprint. This perimeter margin accommodates minor electrode misalignments and ensures that lithium ions encounter anode‐active material rather than the bare current collector, thereby reducing lithium plating and dendrite formation. A 5% overhang was used in this analysis. *C*_a,mat_ and *C*_c,mat_ denote the material‐level practical capacities for anode and cathode in mAh g^−1^. For cathodes, we use practical capacities of 150 mAh g^−1^ for LFP (theoretical capacity: 170 mAh g^−1^), 140 mAh g^−1^ for LCO (274 mAh g^−1^), 200 mAh g^−1^ for NMC811 (257 mAh g^−1^), and 700 mAh g^−1^ for Li_2_S (~60% of 1167 mAh g^−1^) for sulfur‐based systems. *V*_nominal_ is the average discharge voltage of the full cell. For graphite‐based cells, we assume nominal voltage of ~3.2 V for LFP//C, ~3.6 V for LCO//C, 3.7 V for NMC811//C, and ~2.1 V for Li_2_S//C. Replacing graphite with Si reduces the nominal full‐cell voltage by ~0.27. This reduction occurs because the average lithiation potential of Si is higher than that of graphite (~0.40 versus ~0.13 V vs. Li/Li^+^). As a rule of thumb, the nominal cell voltage is approximately the cathode potential minus the anode's potential; therefore, a higher anode potential results in a lower full‐cell voltage. The utilization factor η accounts for the inactive mass (current collectors, separator, electrolyte, casing), which depends on design and form factor—e.g., 75% for high‐energy pouch cell (Volkswagen ID.3) [19] and ~63% for cylindrical NMC//graphite EV cells (mass distribution data from Cerdas et al.) [20]. We use η = 0.70 as a conservative baseline.

Figure 2 shows that graphite‐based cells achieve a maximum energy density of ~315 Wh kg^−1^ with commercial cathodes (maximum with NMC811), and pairing graphite with a sulfur cathode offers no gain, leaving the 500 Wh kg^−1^ milestone out of reach. Introducing a Si anode at a practical capacity of 1070 mAh g^−1^ (~30% of theoretical) raises cell energy to 265 Wh kg^−1^ with LFP (+16% vs. graphite/LFP), 283 Wh kg^−1^ with LCO (+16%), and 395 Wh kg^−1^ with NMC811 (+25%). With sulfur, the same capacity Si delivers ~511 Wh kg^−1^, exceeding the 500 Wh kg^−1^ target. Increasing the utilized Si capacity to 40% of its theoretical value with NMC pushes energy past 400 Wh kg^−1^; however, even an idealized 100%‐capacity Si anode still falls below 500 Wh kg^−1^. Reaching 500 Wh kg^−1^ with intercalation cathodes would require improvement in one or more of the following: cathode capacity, nominal voltage, and system‐level mass utilization efficiency (*η*). Although a value near ~500 Wh kg^−1^ has been reported for specific prototypes, such a value may rely on aggressive reductions in inactive components, including binder, current collectors, and separators, potentially compromising mechanical integrity and safety. By contrast, pairing Si with a sulfur cathode approaches ~700 Wh kg^−1^ under our assumptions and could approach ~800 Wh kg^−1^ if practical sulfur capacity moves toward its theoretical limit.

## 4 Rational Design Strategies for Practical Silicon Anodes

Enabling the commercial viability of Si anodes in LIBs requires addressing several fundamental failure modes. Foremost is the ~300% lithiation‐induced volume expansion, which drives unstable SEI formation, particle fracture and pulverization, and electrode‐level swelling, compromising mechanical integrity and cycle life. At the electrode level, repeated expansion and contraction can induce microcracking, binder fatigue, pore‐structure collapse, and loss of Si–Si, Si–conductive additive, and particle–current collector contact, leading to electrically isolated Si domains, incomplete delithiation, increased impedance, and capacity loss [21]. Binder properties further influence stress distribution and the retention of electrical connectivity, while repeated SEI fracture and regrowth consume cyclable lithium, increase tortuosity, and promote transport heterogeneity across the electrode thickness. Rational electrode design must therefore (i) accommodate volume change to limit fractures and electrode‐level swelling, (ii) preserve mechanical cohesion and electrical connectivity, (iii) shorten Li‐diffusion pathways and improve electronic/ionic transport, and (iv) minimize parasitic Si–electrolyte reactions to stabilize the SEI.

A wide spectrum of architectures has been explored to meet these goals, from particle‐based designs like core–shell and yolk–shell nanoparticles, porous microparticles, and nanoparticle‐derived microspheres (hereafter, secondary microspheres) to structured electrodes such as Si nanowires and thin films on high‐aspect‐ratio scaffolds. However, to fully assess the commercial viability of Si anodes, several key metrics must be considered in half‐cell evaluations, including practical capacity (mAh g^−1^), mass loading (mg cm^−2^), areal capacity (mAh cm^−2^), initial Coulombic efficiency (ICE), cycling stability, long‐term CE, tap density (g cm^−3^), and volumetric capacity (mAh cm^−3^). Gravimetric capacity (mAh g^−1^) dictates the potential for cell‐level energy beyond graphite; to be impactful, practical Si capacities ≥1400 mAh g^−1^ are generally needed, as discussed earlier. ICE, which reflects first‐cycle Li loss to SEI and other irreversible reactions, is crucial for full‐cell viability. Porous Si structures, despite their ability to accommodate expansion, often exhibit lower ICE due to their high surface area, necessitating strategies like prelithiation or surface modifications to improve lithium retention.

Areal capacity (mAh cm^−2^) is another critical metric for practical Si‐anode evaluation because it determines the amount of charge stored per unit electrode area and directly impacts cell‐level energy density. Areal capacity is determined by both gravimetric capacity and mass loading according to: areal capacity (mAh cm^−2^) = mass loading (mg cm^−2^)  × gravimetric capacity (mAh g^−1^)/1000. While many reports emphasize high gravimetric capacities, these values are often obtained at relatively low mass loadings and may not translate into commercially relevant electrode performance. In practical lithium‐ion cells, cathode areal capacities are typically in the range of ~3–4 mAh cm^−2^, requiring Si anodes to achieve comparable areal capacities while maintaining appropriate N/P ratios. Therefore, mass loading should be considered alongside gravimetric capacity when assessing the practical viability of Si anodes. To facilitate such comparisons, mass loading values are included in the summary tables throughout this review.

**FIGURE 3:**
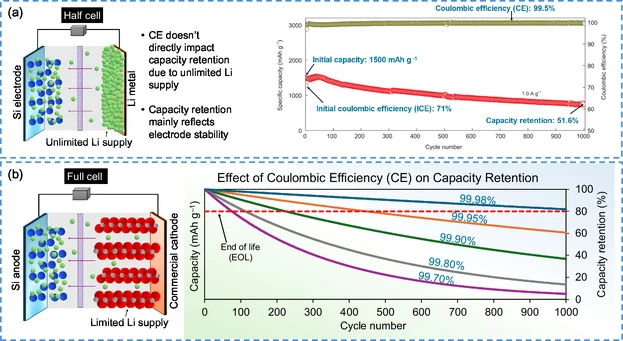
Comparison of half‐cell versus full‐cell performance: (a) half‐cell performance [22] showing that capacity retention mainly reflects electrode stability, with CE having minimal effect. Reproduced with permission [22]. Copyright 2021, Oxford University Press. (b) Impact of CE on full‐cell capacity retention, highlighting the need for ∼99.98% CE to achieve 1000–cycle practical battery life.

Cycling stability and CE provide complementary views of durability. Cycling stability—evaluated by capacity retention as a function of cycle number in half cells—primarily reflects electrode integrity, revealing whether fracture, electrical isolation, or other degradation mechanisms cause active material loss during repeated cycles. CE—the ratio of discharge to charge capacity per cycle—is a key measure of SEI stability and parasitic surface reactions. In half‐cells, where Si is paired with Li metal, Li^+^ ions consumed by parasitic reactions or trapped within the anode can be replenished by the Li‐metal counter electrode in subsequent cycles; consequently, poor CE may have only a limited impact on apparent capacity retention. For example, the Si anode shown in Figure 3a exhibits a CE of 99.5% yet retains 51.8% of its initial capacity after 1000 cycles, illustrating that half‐cell capacity fade is often governed by electrode degradation rather than lithium inventory loss. However, the measured half‐cell CE remains a critical predictor of full‐cell performance. In full cells, where lithium inventory is fixed by the cathode, lithium lost to SEI growth or trapped within the anode cannot be replenished, causing CE losses to accumulate directly as capacity fade (Figure 3b) [23]. Achieving 1000 full‐cell cycles typically requires a CE of ~99.98%, while 500 and 250 cycles demand >99.95% and 99.9%, respectively, with values below 99.8% being generally impractical. Therefore, precise and explicit CE reporting in half‐cell studies is essential for credible full‐cell projections.

**FIGURE 4:**
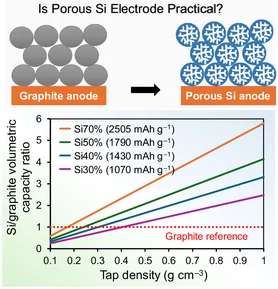
Practicality of porous Si anodes: volumetric capacity comparison at varying porosities, with Si/graphite ratios >1 indicating improved performance.

Volumetric performance is equally important for commercial viability. Tap density (mass per packed volume after tapping), *ρ*_tap_, governs volumetric capacity: $C_{\mathrm{vol}}$ (mAh cm^−3^) = $\rho_{\mathrm{tap}}$ (g cm^−3^) $\times \;C_{g}$ (mAh g^−1^). Several reviews [24, 25, 26] have questioned whether nano/porous Si can meet the required volumetric metrics because their tap density is inherently low and decreases with increasing porosity: *ρ*_tap_ = $\rho_{\text{solid}}$ (1 − ϕ), where $\rho_{\text{solid}}$ is the intrinsic density (Si ≈ 2.33 g cm^−3^; graphite ≈ 2.26 g cm^−3^) and ϕ is the porosity. Commercial natural‐graphite anode powders typically show $\rho_{\mathrm{tap}}$ ≈ 1.1–1.2 g cm^−3^ [27], corresponding to ϕ ≈51%–47%. Using graphite with $\rho_{\mathrm{tap}}$ = 1.2 g cm^−3^ as a benchmark, we evaluated the Si/graphite volumetric‐capacity ratio for Si anodes delivering 30%−70% of theoretical capacity across a range of $\rho_{\mathrm{tap}}$ values (Figure 4). To be commercially relevant, Si must match or exceed graphite's volumetric capacity, i.e., the ratio should be ≥1. Figure 4 shows that silicon can match graphite's volumetric capacity even at relatively low tap densities: ~0.4 g cm^−3^ at 1070 mAh g^−1^, ~0.3 g cm^−3^ at 1430 mAh g^−1^, ~0.24 g cm^−3^ at 1790 mAh g^−1^, and 0.17 g cm^−3^ at 2505 mAh g^−1^. These thresholds highlight that volumetric performance depends on co‐optimizing tap density and specific capacity. Accordingly, low‐tap‐density Si should not be dismissed, as it can remain competitive when sufficient capacity is achieved.

In this review, we critically analyze recent advancements in Si‐only electrodes (silicon with an optional thin passivation coating) and Si‐dominant electrodes in which silicon supplies most of the reversible capacity in a carbon/porous host. While graphite‐Si composite [28] anodes represent an important current industrial approach for incorporating silicon into commercial Li‐ion batteries, achieving cell‐level energy densities approaching 500 Wh kg^−1^ remains challenging with graphite‐dominant Si blends because the overall capacity is constrained by the graphite‐rich electrode composition. Therefore, this review focuses on Si‐only and Si‐dominant anode architectures that are more directly relevant to high‐energy cells targeting ~500 Wh kg^−1^. We organize the discussion into two parts:

1. Particle‐based Si anodes
  • Si nanoparticles (SiNPs): core‐shell and yolk‐shell SiNPs, and secondary microspheres from SiNPs
  • Si microparticles (µ‐Si): dense and porous (μ‐Si)
2. Structured Si anodes
  • Si nanowires
  • Si thin films on 3D scaffolds

Throughout, we focus on electrode‐engineering strategies for Si‐only and Si‐dominant anodes—specifically, volume‐buffering designs and thin surface passivation. Binder designs, electrolyte formulation, cost analysis, and manufacturing scale‐up are outside the scope of this review, though each is important for practical deployment. We evaluate half‐cell performance against practical capacity (mAh g^−1^), ICE, long‐term CE, cycling stability, and tap density to assess commercial relevance and identify routes to practical implementation.

### 4.1 Particle‐based silicon anodes

#### 4.1.1 Silicon nanoparticles

Si nanoparticles (SiNPs) are widely studied because downsizing mitigates a primary failure mode of bulk Si—fracture driven by ~300% lithiation‐induced expansion. Below a critical size, Si can accommodate strain without developing destructive stresses, preserving particle integrity during cycling. Crystallinity strongly influences this behavior: crystalline and amorphous Si exhibit distinct lithiation pathways, expansion symmetry, and fracture thresholds.

**FIGURE 5:**
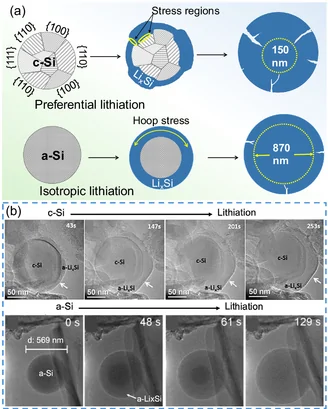
Critical fracture limits of amorphous and crystalline SiNPs: (a) anisotropic versus isotropic lithiation‐induced fracture. (b) TEM images of c‐Si [33] and a‐Si [34] lithiation, showing no cracking within the critical size limit. Reproduced with permission [33]. Copyright 2014, Springer Nature. Adapted with permission [34]. Copyright 2013, ACS.

Experiments show that crystalline Si (c‐Si) particles below a critical diameter of ~150 nm can tolerate lithiation strain without cracking [29]. Lithiation is anisotropic and facet‐dependent, arising from variations in lithium diffusion kinetics across different crystal facets. First‐principles simulations [30] and experimental studies [31, 32] indicate that the (110) surface is energetically more favorable for lithiation than the (100) or (111) surfaces. As a result, lithium insertion proceeds more rapidly along the (110) plane, leading to preferential expansion in this direction (Figure 5). This nonuniform expansion induces strain at the boundaries of different crystal planes, triggering crack initiation and propagation. Furthermore, lithiation of crystalline Si forms an amorphous Li_*x*_Si shell around an unlithiated c‐Si core. The moving a‐Li_*x*_Si/c‐Si interface concentrates tensile stress and further promotes fractures, especially in larger particles. In contrast, amorphous Si (a‐Si) exhibits greater fracture resistance, with a higher critical size (~870 nm) [34] before cracking is observed. Lithiation is more isotropic, distributing strain more uniformly. When failure occurs, it is typically governed by hoop stress: as Li is inserted, the outer layers experience tensile stress that eventually nucleates circumferential cracks (Figure 5).

Despite improved fracture tolerance, SiNPs face several challenges. One major issue is the agglomeration of particles [35, 36], driven by the high surface energy of SiNPs, which causes them to cluster together. This agglomeration reduces the electrochemically active surface area and disrupts lithium transport kinetics, leading to inhomogeneous lithiation and localized stress buildup that accelerate electrode degradation. Additionally, the large specific surface area of SiNPs (~100 m^2^ g^−1^) increases electrolyte‐exposed surface, amplifying side reactions and causing excessive SEI growth, lithium consumption, low ICE, and continued capacity loss.

To address these challenges, various surface engineering architectures have been used, primarily core–shell and yolk–shell structures, where the core/yolk consists of Si nanoparticles and the shell is typically composed of carbon, polymer, metal oxides such as TiO_2_ and SiO_2_, or their hybrids. In core–shell structures, the Si core is directly encapsulated by the shell, providing a protective barrier that enhances stability and conductivity while mitigating surface reactions. In contrast, yolk–shell architectures introduce a deliberate void between the Si “yolk” and the outer shell, allowing the core to expand freely during lithiation and thereby reducing mechanical damage. These SiNP‐based architectures and their reported practical metrics, including mass loading, ICE, CE, cycling conditions, and capacity retention, are summarized in Table 1.

**TABLE 1:** Summary of reported silicon nanoparticle (SiNP)‐based anodes, including core–shell, yolk–shell, core–dual shell, and secondary microsphere architectures.

References	Structure	Tap density, g cm^−3^	Mass loading, mg cm^−2^	Current density, A g^−1^	Initial capacity, mAh g^−1^	ICE, %	CE, %	Cycles	Capacity retention, %
Core–shell SiNP									
[37]	Si@10C	—	—	0.5	3394	74.9	99.5	500	30
[38]	Si@C	—	1.02	0.2	1728	—	99*	100	68
[39]	Si@NC	—	0.9	0.5	2273	88.9	99.77	300	42
[40]	Si@TiO_2_	—	—	0.4	3580.8	80.8	97.97	50	44
Yolk–shell SiNP									
[41]	Si@C	—	—	0.1	783	—	—	100	98
[42]	Si@CNi	—	2−3.5	0.3	1382	60	99.3	600	95
[43]	Si@V_3_O_4_@C	—	0.5–0.7	0.5	1517	66.8	99.3*	700	70
Core‐dual‐shell SiNP									
[44]	Si@C@TiO_2_	—	1.5−2.1	0.42	2390	73	98	710	42
[45]	Si@a‐TiO_2_@a‐C	—	—	1	~1500	87.3	—	500	59
[46]	Si@SiO_*x*_@C		0.27	4	~2000	91	99.4	500	63
[47]	Si@SiO_*x*_@C	—	2.04	1	1390	82.4	—	1000	50
[48]	Si@SiO_*x*_@CBPOD	—	1	4	1675	88.2	—	1000	64
Secondary microsphere									
[49]	Si@TiO_2_@C (SA‐SiTC)	0.68	1.7	2	~1300	80.9	99.8*	1000	65
[50]	P‐Si/C@C	0.92	3.5	1	813.5	89.8	—	820	87
[51]	D‐Si@RF‐CTP	0.86	0.7	0.5	1007.5	78.6	—	200	80
[52]	Si NPs cluster@Si@G	0.79	0.6	2.1	~2000	90.4	99.7*	300	70
[53]	VGSs‐YS‐Si/C	—	1.5–1.8	4.2	~1200	88.3	99.89*	1000	80

* denotes average Coulombic efficiency (CE).

i. Core–shell and yolk–shell (YS) SiNPs

**FIGURE 6:**
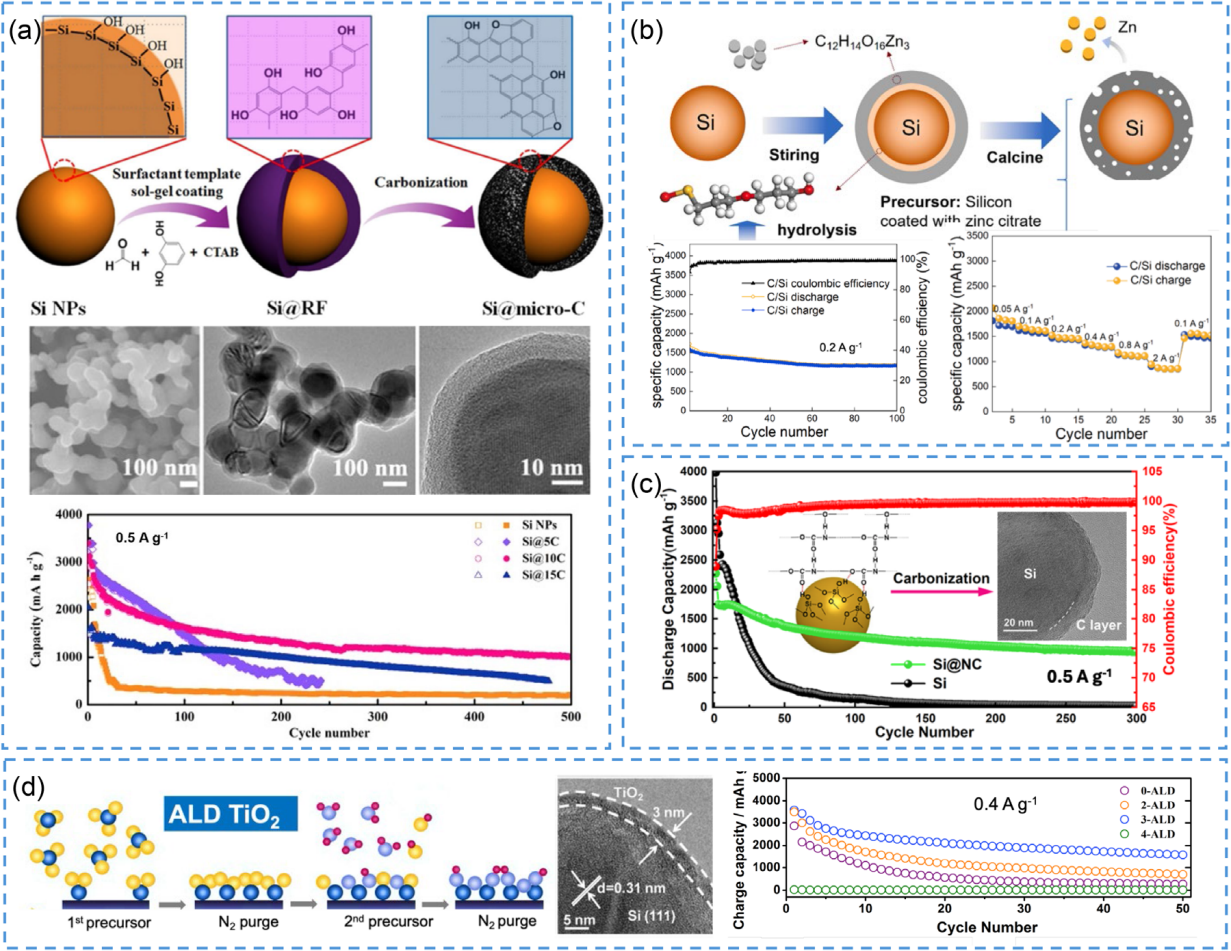
Core–shell SiNP‐based anodes with various shell materials: (a) fabrication of microporous carbon shell, SEM/TEM images and cycling performance. Reproduced with permission [37]. Copyright 2016, Elsevier. (b) Mesoporous carbon shell fabrication, cycling stability, and rate performance. Adapted with permission [38]. Copyright 2024, Elsevier. (c) Performance of N‐doped carbon shell. Reproduced with permission [39]. Copyright 2024, ACS. (d) ALD‐deposited TiO_2_ shell with cycling performance for various coating thicknesses. Adapted with permission [40]. Copyright 2016, Elsevier.

To stabilize SiNPs, most core–shell designs employ carbon or TiO_2_ shells (Figure 6). Wei et al. [37] varied microporous carbon thickness (Figure 6a): a 5 nm carbon layer (Si@5C) failed to accommodate cycling strain and delivered poor cycling performance, whereas Si@10C, with its microporous structure and optimized diffusion pathways, sustained 1006 mAh g^−1^ with >99.5% CE after 500 cycles at 0.5 A g^−1^. While ICE improved slightly from 72.6% (bare Si) to 74.9% (Si@10C), capacity continued to fade during cycling, likely due to electrode swelling and microcracking. Additionally, it has been reported that carbonization temperature influences the elasticity and conductivity of the carbon coating [54], as carbon exists in two different bonding forms—sp^3^ (diamond‐like, strong but less conductive) and sp^2^ (graphene‐like, conductive)—each affecting performance differently. Achieving an elastic, robust, and conductive carbon coating requires carefully balancing the sp^3^ and sp^2^ carbon content. Mesoporous carbon (~4.5 nm pores) has also been explored (Figure 6b) [38], which showed good capacity and rate capability, retaining 1170 mAh g^−1^ at 0.2 A g^−1^ after 100 cycles and 944 mAh g^−1^ at 2 A g^−1^, with ~99% average CE. However, the CE remains below stringent full‐cell needs, and extended cycling is required. Additionally, N‐doped carbon [39] (Figure 6c) has been explored for its ability to enhance ion diffusion and provide a pseudocapacitive contribution. It increases ICE from 75.85% (Si) to 88.89% (Si@NC), with the Si@NC electrode maintaining 945.63 mAh g^−1^ and a CE of 99.77% after 300 cycles. Overall, Si@C core–shells buffer expansion to some extent but, owing to limited shell stiffness, often allow electrode‐level swelling and microcracking, as observed in multiple studies [37, 55, 56], resulting in poor capacity retention during cycling. Mitigating swelling and associated microcracking remains a key design target.

TiO_2_ coatings have gained attention for their mechanical strength and ability to suppress volume expansion [55]. Unlike SiO_2_ and SnO_2_, which undergo volume expansions of approximately 100% and 300% upon lithiation, TiO_2_ experiences less than 4% expansion. This minimal expansion makes TiO_2_ an effective material for buffering volumetric changes. TiO_2_ shells from atomic layer deposition (ALD) [40] and sol–gel routes [57] have been demonstrated for Si@TiO_2_ core–shell structures. ALD yields a uniform shell (TEM‐verified) that helped achieve a capacity of 1580.3 mAh g^−1^ after 50 cycles (Figure 6d), but with a low CE (~97.97%) [40]. In sol–gel systems, amorphous TiO_2_ outperformed crystalline TiO_2_, consistent with higher elasticity in the amorphous phase [57]. Nonetheless, many Si@TiO_2_ core–shells struggle in long‐term cycling, likely because repeated Si expansion fractures the rigid shell, leading to electrode‐level swelling and cracking. In this context, ALD Al_2_O_3_/TiO_2_ nanolaminates (sub‐nm periods, controlled nonstoichiometry) provide a promising design to balance Li^+^ permeability and mechanical robustness, potentially exceeding the thickness limits of single‐oxide shells [58].

**FIGURE 7:**
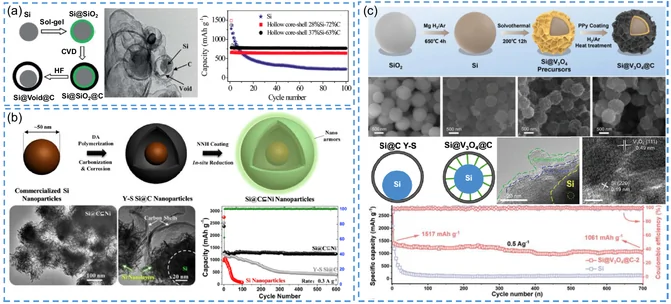
Yolk–shell SiNP anodes and void‐filling strategies: (a) Si@Void@C synthesis, TEM imaging, and cycling performance. Adapted with permission [41]. Copyright 2014, RSC Pub. (b) Encapsulating Y‐S Si@C nanoparticles with tough and malleable Ni nanoarmors (Si@C⊆Ni). Reproduced with permission [42]. Copyright 2018, ACS. (c) V_3_O_4_ sheets integration to enhance contact between the carbon shell and Si yolk. Adapted with permission [43]. Copyright 2023, Wiley.

To overcome the limitations of core–shell structures, yolk–shell designs, particularly Si@C, have been explored (Figure 7). This approach introduces an internal gap so the Si “yolk” can expand without stressing the outer shell, thereby mitigating electrode‐level swelling. A common route to fabricate a yolk–shell structure is to first coat SiNPs with a sacrificial SiO_2_ layer, deposit carbon, and then etch the SiO_2_ to yield a Si@Void@C architecture. Huachao et al. [41] implemented this strategy with a 37% Si–63% C nanocomposite, which exhibited an initial charge capacity of 783 mAh g^−1^ and retained 98% of its capacity after 100 cycles (Figure 7a). However, the overall capacity remains limited due to the higher carbon content. To reinforce the carbon shell in Si@C yolk–shell, malleable Ni nanoarmors [42] were introduced (Figure 7b). This design effectively restrained volume expansion, mitigated aggregation, and improved conductivity by the nanoarmors acting as nanoscale current collectors. As a result, Si@C⊆Ni demonstrated excellent cycling stability, maintaining a capacity of ~1307 mAh g^−1^ with no measurable decay over 600 cycles. Despite a low ICE (~60%), CE always remained above ~99.3%, but further improvement is needed for full‐cell viability.

In pure yolk–shell Si/C anodes, limited point‐to‐point contact between the core and shell hinders ion and electron transport, potentially limiting capacity. To overcome this, Ziyang et al. [43] incorporated V_3_O_4_ sheets within the voids via a solvothermal reaction. This design bridges the void between the carbon shell and Si core, improving electron percolation and Li^+^ transport (Figure 7c). As a result, the optimized electrode demonstrates a high capacity of 1517 mAh g^−1^, retaining 70% capacity over 700 cycles with an average CE of 99.3%. This underscores the need for strategic void filling—maintaining enough space to buffer volume expansion while ensuring sufficient contact for efficient ion and electron transport.

**FIGURE 8:**
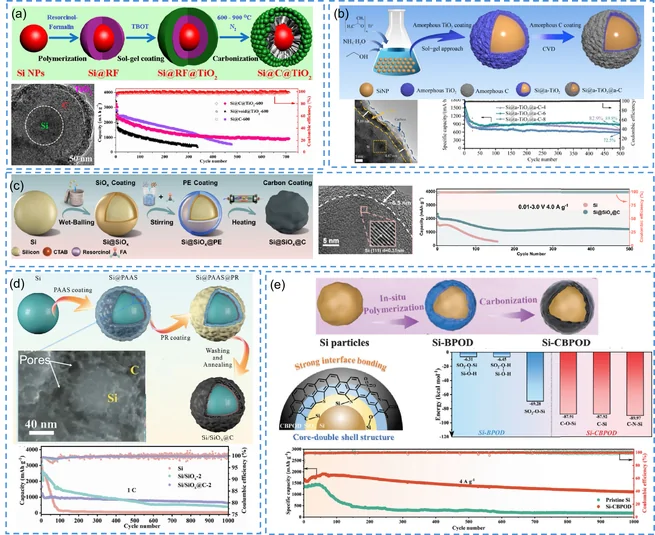
Core dual‐shell structures SiNPs: (a) Si@C@TiO_2_ synthesis via a two‐step sol–gel process. Reproduced with permission [44]. Copyright 2016, ACS. (b) Si@a‐TiO_2_@a‐C synthesized via sol–gel and CVD. Adapted with permission [45]. Copyright 2024, Springer Nature. (c) Si@SiO_*x*_@C dual‐shell structure. Reproduced with permission [46]. Copyright 2024, Elsevier. (d) Introduction of voids in the Si@SiO_*x*_@C. Reproduced with permission [47]. Copyright 2024, Wiley. (e) Porous N/S co‐doped carbon layer with a SiO_*x*_ inner shell. Reproduced with permission [48]. Copyright 2024, Wiley.

To leverage the synergistic effect of a conductive, elastic carbon layer with a mechanically stable oxide layer, core‐dual‐shell structures have gained significant attention (Figure 8). The Si@C@TiO_2_ structure [44], featuring mesoporous carbon and crystalline TiO_2_ as dual shells, delivered 1726 mAh g^−1^ over 100 cycles and retained 1010 mAh g^−1^ after 710 cycles at 0.42 A g^−1^, although its overall retention remained suboptimal (Figure 8a). A similar design has been reported recently with only 100‐cycle data, requiring longer‐term validation [59]. In a reverse Si@TiO_2_@C configuration, cycling stability was poor, with rapid capacity loss within 25 cycles, as shown in Figure 8b [45]. These data underscore a strong ordering effect.

Replacing TiO_2_ with SiO_2_ in dual‐shell structures has shown promising results. For example, Si@SiO_*x*_@C dual‐shell structure [46] was synthesized using ball milling and phenolic resin‐assisted encapsulation, to leverage the synergistic effects of SiO_*x*_ layer for enhanced Li^+^ transport/expansion mitigation and carbon layer for conductivity and interfacial passivation (Figure 8c). This design achieved a high ICE of 91%, retained 1250 mAh g^−1^ after 500 cycles at 4 A g^−1^, and maintained an average CE of over 99.4%. In a similar design, a recent study introduced intentional pores/space into the Si@SiO_*x*_@C structures (Figure 8d) [47]. This structure delivered 685.3 mAh g^−1^ after 1000 cycles at 1 C, with ICE of 82.4%. Although the cycling stability appears promising, the modest capacity likely reflects limited transport across the pores. Notably, the capacity drop appears only after adding the outer C coating; Si@SiO_*x*_ alone shows higher capacity. The same trend appears in Si@SiO_2_@void@C [60] composite that delivered only ~500 mAh g^−1^ at 1 A g^−1^.

To further enhance the outer carbon layer, nitrogen/sulfur co‐doped porous carbon (CBPOD) has been used as the outer shell in a Si@SiO_*x*_@CBPOD architecture (Figure 8e) [48], which delivered 1110.8 mAh g^−1^ after 1000 cycles at 4 A g^−1^ with a higher ICE of 88.2%. The higher ICE likely reflects N/S co‐doping modifying the carbon shell's surface chemistry: substitutional N and S reduce strong Li‐binding defects/edge sites, lowering irreversible Li loss at the carbon interface. Given that commercial graphite achieves ~90%–94% ICE, additional optimization remains necessary.

More recently, metal–organic framework (MOF)‐derived architectures have extended the dual‐shell concept beyond conventional coating designs. Using a ZIF‐67‐derived porous carbon framework, Si nanoparticles were integrated with a CoSi_2_ alloy interface and outer graphene shell to form a gradient multi‐barrier architecture [61]. By collectively confining multiple Si nanoparticles within a conductive carbon matrix, the design improved stress dissipation, electrical conductivity, and interfacial stability, achieving an ICE of 88.9% and retaining 1104.3 mAh g^−1^ after 300 cycles at 0.5 A g^−1^. These results further support the importance of reducing direct electrolyte exposure and stabilizing the Si/electrolyte interface through hierarchical confinement strategies.

Recent studies have also moved beyond simple core–shell designs by embedding SiNPs into conductive porous networks. For example, rGO‐modified porous TiO_2_ frameworks [62] and GaIn‐decorated porous carbon frameworks [63] have been used to improve ion/electron transport, buffer volume expansion, and stabilize the SEI. Although these architectures improve cycling stability, their moderate CE values indicate that further reduction of electrolyte‐exposed surface area and better interfacial control are still needed for practical long‐cycle cells.

While modified yolk–shell structures with void fillers and core‐dual‐shell SiNPs have demonstrated promising electrode stability over 500 cycles, their practical viability remains constrained by low CE, as their high electrolyte‐exposed surface area accelerates SEI formation and irreversible Li consumption. A promising route is to assemble SiNPs into dense secondary microspheres. By consolidating many nanoparticles into a single granule with a thin passivation shell and controlled internal porosity, the electrolyte‐exposed area drops sharply, limiting SEI formation, reducing first‐cycle Li loss, and suppressing ongoing parasitic reactions. Denser packing raises tap/electrode density, improving volumetric capacity—a key requirement for commercial viability.

ii. Secondary microspheres assembled from SiNPs

Secondary microspheres—micron‐scale assemblies of SiNPs often embedded in a conductive carbon matrix—preserve the kinetic and fracture tolerance of nano‐Si within a high‐density, manufacturable morphology. By lowering specific surface area and suppressing agglomeration, they mitigate parasitic SEI growth and raise ICE; their higher tap density improves volumetric capacity and enables effective electrode densification during calendering. Engineered internal porosity/voids preserve short Li‐diffusion paths and accommodate expansion, while the carbon matrix provides electronic paths and interfacial passivation. Together, these attributes position secondary microspheres as a practical platform for Si‐dominant anodes in slurry‐cast LIB electrodes.

**FIGURE 9:**
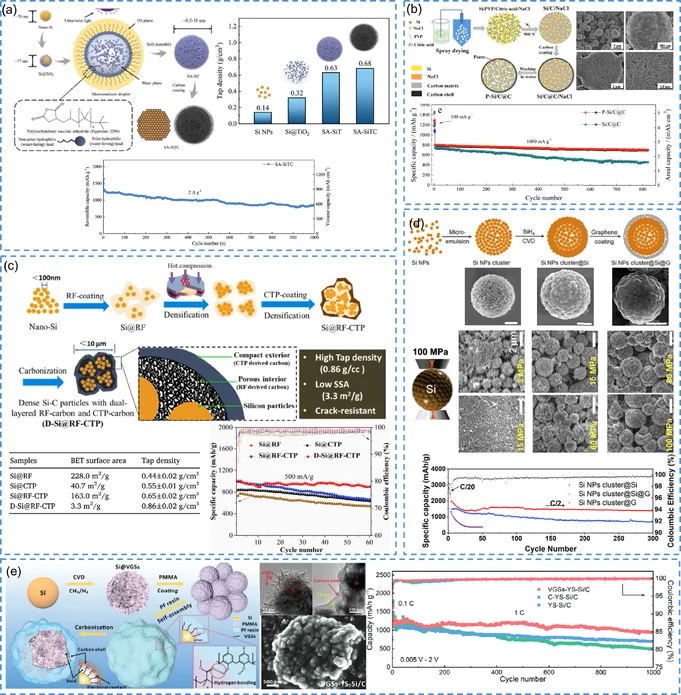
Secondary microspheres derived from SiNPs: (a) self‐assembled hierarchical Si@TiO_2_@C (SA‐SiTC) microspheres from core–shell Si@TiO_2_ building blocks. Reproduced with permission [49]. Copyright 2020, Wiley. (b) Pore‐rich Si/C microspheres (P‐Si/C@C) with SiNPs immobilized in a cross‐linked C matrix, synthesized via spray‐drying and CVD. Reproduced with permission [50]. Copyright 2022, ACS. (c) Si particles embedded in a dual‐layer carbon matrix with a porous interior and compact exterior. Reproduced with permission [51]. Copyright 2023, Elsevier. (d) Pressure‐resistant Si microspheres with a dense Si shell and graphene coating. Adapted with permission [52]. Copyright 2018, ACS. (e) Hierarchical VGSs‐YS‐Si/C microparticles with vertical graphene sheets for enhanced Si=C connectivity [53]. Copyright 2018, Wiley.

In this regard, Jiewen et al. [49] prepared a self‐assembled hierarchical Si@TiO_2_@C microsphere architecture (Figure 9a): core–shell Si@TiO_2_ nanoparticles were first synthesized via a sol–gel route, then self‐assembled into microspheres in a 1‐octadecene solution, and finally carbonized to deposit a conformal carbon shell. This structure significantly improved tap density (0.68 vs. 0.14 g cm^−3^ for SiNPs) and delivered 842.6 mAh g^−1^ after 1000 cycles at 2 A g^−1^ (65% retention). Similarly, a spray‐dried, CVD‐coated Si/CNT/GO@C microsphere [64] reached a high capacity (2921 mAh g^−1^) but showed modest cycling stability (1542 mAh g^−1^ after 100 cycles). In a related spray‐drying/CVD strategy, pore‐rich Si/C microspheres (P‐Si/C@C) were fabricated (Figure 9b) [50]. NaCl was used as a removable template during microsphere assembly and subsequently leached with water, creating internal voids without harsh chemical etching. Despite the added porosity, tap density remained high (0.92 g cm^−3^), resulting in volumetric capacity of 1168 mAh cm^−3^ at 100 mA g^−1^. In addition, the electrode swelling was limited to 18.1%, and the electrode retained 87% capacity after 800 cycles with ICE of 89.8%, although the gravimetric capacity remained <800 mAh g^−1^. Additionally, a dual‐layer carbon matrix [51] with a compact exterior and porous interior was developed (Figure 9c): the dense outer layer limits electrolyte ingress, while the porous interior accommodates Si expansion. With a high tap density (0.86 g cm^−3^) and low surface area (3.3 m^2^ g^−1^), the electrode retained ~80.3% capacity over 200 cycles at 0.5 A g^−1^.

For compatibility with existing manufacturing processes, electrodes must endure high‐pressure calendering. This necessitates mechanically robust secondary particles. In this context, Jiangyan et al. [52] developed a pressure‐resistant Si structure by coating micrometer‐sized Si clusters with a dense Si shell and a graphene cage (tap density of 0.79 g cm^−3^) that survived calendering pressures (Figure 9d). Pressure tests showed uncoated Si clusters collapsed at 15 MPa, while those with a 200 nm Si shell remained intact at 100 MPa. This design achieved CE > 99.5%, capacity of 1388 mAh g^−1^ after 300 cycles at 2.1 A g^−1^, and only 13.7% volume expansion. However, an initial capacity drop was observed, as shown in Figure 9d, which suggests stress‐induced electrical connectivity disruption within the inner cluster. Incorporating a robust conductive network (e.g., CNTs/graphene) may mitigate this issue. Building on this, a recent study synthesized a hierarchical VGSs‐YS‐Si/C anode [53], where vertical graphene sheets (VGSs) were first grown on Si nanoparticles via CVD, followed by PMMA encapsulation to form a yolk–shell (YS) structure. Polymer self‐assembly and phenolic resin coating were then applied, followed by one‐step carbonization to obtain VGSs‐YS‐Si/C microparticles (Figure 9e). This design enhances electrochemical and mechanical stability by establishing strong connections between the Si core and carbon shell through VGSs. As a result, VGSs‐YS‐Si/C achieved 1683.2 mAh g^−1^ at 0.1 C, and capacity retention of 80.1% after 1000 cycles at 1 C, with impressive ICE of 88.3% and 99.89% average CE (stabilizing > 99.50% within five cycles). Compressive strength tests confirmed its structural integrity under 200 MPa, exceeding industrial electrode pressure requirements.

Overall, secondary microspheres are a credible path to commercial Si anodes: they raise tap density, improve ICE, sustain high long‐term CE with stable cycling, and withstand industrial calendering. What remains is a single architecture that co‐delivers ICE ≥90%, long‐term CE ≳99.90%, and ≥1000 mAh g^−1^ at high tap density with low swelling.

#### 4.1.2 Silicon microparticles (µ‐Si)

Micrometer‐scale Si (μ‐Si) particles are attracting interest because they offer higher tap density, much lower specific surface area, and lower production costs relative to SiNPs. Because specific surface area decreases with particle size, µ‐Si exposes far less electrolyte‐facing area than SiNPs (for the same mass), which reduces SEI area and initial lithium loss, improving ICE. However, μ‐Si anodes have familiar drawbacks: large volume change during lithiation drives cracking/pulverization, and longer electron/Li^+^ transport paths hinder kinetics. Two complementary design routes have emerged: (i) dense μ‐Si with encapsulation/coatings to maintain electrical contact even after fracture, and (ii) porous μ‐Si architectures that accommodate expansion, typically combined with surface passivation to stabilize the interface and improve conductivity. These μ‐Si architectures and their reported practical metrics, including tap density, mass loading, ICE, CE, cycling conditions, and capacity retention, are summarized in Table 2.

**TABLE 2:** Summary of reported micro‐silicon (μ‐Si) anodes, including dense and porous μ‐Si architectures.

References	Structure	Tap density, g cm^−3^	Mass loading, mg cm^−2^	Current density, A g^−1^	Initial capacity, mAh g^−1^	ICE, %	CE, %	Cycles	Capacity retention, %
Bulk μ‐Si									
[65]	SiMP@Gr	—	0.8	2.1	1647	93.2	99.9	300	85
[22]	SiMP@C‐GN	—	1.0–2.3	1	1500	82.6	99.5*	500	70
[66]	Si/LM@C‐CNF	4.15	1.5	2	~1700	87.4	99.4*	400	48
[67]	Si^(0)^@SiO_2_^(v)^@C	—	0.67	2	~1000	83	—	1500	86
Porous μ‐Si									
[68]	PSi/SCNT‐SRGO	0.42	0.6	1	1688	81.1	—	600	60
[69]	P−Si@C	2.3	0.8–0.9	1	866	59.3	99.35*	150	98
[70]	PSi@PPy‐Fe	—	—	1	2044	77.7	—	200	91
[71]	m‐GPSi	—	0.9–1.0	0.1	3134	90	99.2*	100	80

* denotes average Coulombic efficiency (CE).

i. Dense µ‐Si

**FIGURE 10:**
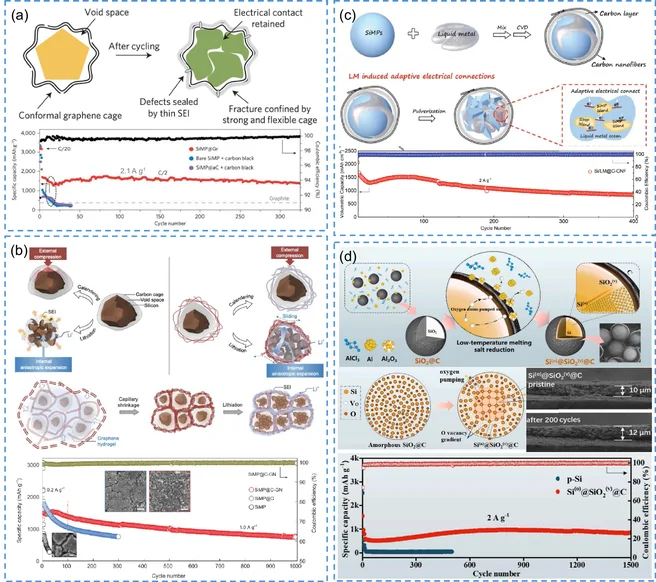
Bulk μ‐Si anodes with various encapsulation strategies: (a) multilayer graphene cages conformally synthesized around Si microparticles. Reproduced with permission [65]. Copyright 2016, Springer Nature. (b) SiMP@C‐GN, featuring a carbon cage (“cell membrane”) reinforced by a dense and robust graphene shell (“cell wall”), inspired by the structural stability of plant cells [22]. Copyright 2021, Oxford University Press. (c) Liquid metal (LM) encapsulation to form an adaptive conducting network, reconnecting pulverized Si fragments. Reproduced with permission [66]. Copyright 2022, Wiley. (d) Oxygen pumping‐assisted microsphere, creating a coherent structure from crystalline Si core to disordered SiO_2_ shell. Reproduced with permission [67]. Copyright 2025, Elsevier.

Dense μ‐Si is often encapsulated with graphene, owing to its strength, flexibility, and low defect density. Yuzhang et al. [65] grew graphene on Ni‐coated μ‐Si via a dissolution‐precipitation mechanism, where Ni served as both a catalyst and a sacrificial layer. After Ni etching, a ~10 nm graphene cage physically confined Si, buffering expansion and trapping fragments (Figure 10a). The anode showed an ICE of 93.2%, while its CE rose to 99.5% by 5 cycles and 99.9% by 10 cycles. It delivered ~3300 mAh g^−1^ at 0.2 A g^−1^, and >85% retention after 300 cycles at 2.1 A g^−1^. However, closer inspection of the cycling plot shows a steep drop in the first ~20 cycles, suggesting possible electrical isolation due to particle fractures. Even so, ICE is high—comparable to graphite. This aligns with a graphite‐like interface: the stacked, low‐defect graphene shell presents few Li‐trapping sites, minimizing first‐cycle loss. This encapsulation strategy may be extended to other Si morphologies to raise ICE. Later, a plant‐cell‐inspired SiMP@C‐GN architecture [22] was explored by capillary‐driven shrinkage of a graphene hydrogel around μ‐Si, followed by precise inner‐void tailoring, yielding a strong yet ductile carbon cage (Figure 10b). It achieved a CE of 99.5% but only ~70% capacity retention (≈1050 mAh g^−1^) after 500 cycles at 1 A g^−1^.

While encapsulation methods show promising performance, they may not fully maintain electrical connectivity between fractured Si particles. To address this, eutectic gallium–indium (EGaIn) liquid metal (LM) [66] has been introduced as a self‐healing conductor that reconnects fractured Si within a confining carbon shell (Figure 10c). The concept demonstrates feasibility, but longer‐term stability and practicality remain to be established. A distinct gradient architecture, Si^(0)^@SiO_2_^(v)^@C [67], formed via oxygen pumping in low‐temperature molten‐salt reduction (250 °C), yields a crystalline‐Si core with a disordered SiO_2_ shell and a final carbon overcoat, adding voids between SiO_2_ and C after Al reduction/acid treatment (Figure 10d). It delivered ~84.6% capacity retention after 1500 cycles at 2 A g^−1^ with an ICE of 83%. The results are promising; however, CE was not explicitly reported—a critical metric for projecting full‐cell durability.

Despite their higher tap density and reduced side reactions, dense μ‐Si particles often show only modest gains in specific capacity, likely due to incomplete lithiation caused by long Li‐ion diffusion lengths and loss of electrical contact as particles fracture. These limitations have motivated the development of porous μ‐Si designs.

ii. Porous µ‐Si

Porous μ‐Si can be synthesized using different methods depending on the starting material: magnesiothermic reduction of SiO_2_, metal‐assisted chemical etching (MACE) of bulk Si, or chemical dealloying of Si–metal alloys. In magnesiothermic reduction, Mg reduces SiO_2_ to Si at ~600–700 °C, and reaction by‐products are acid‐leached, yielding porous Si. In MACE, a thin Ag/Au catalyst drives localized dissolution of Si in HF/H_2_O_2_, “drilling” oriented pores set by catalyst loading and etch time. In chemical dealloying, selective leaching of the metal from a Si–M alloy (e.g., Si–Al) leaves an interconnected porous Si skeleton governed by the alloy microstructure and leach conditions. While porosity mitigates expansion, it raises surface area, decreasing ICE and destabilizing the SEI unless passivated.

**FIGURE 11:**
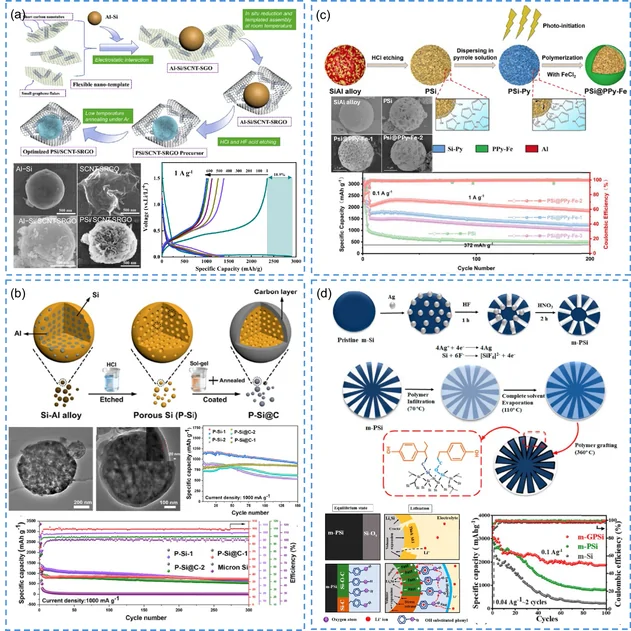
Porous μ‐Si anodes with various coating strategies: (a) weave cage‐like carbon nanostructure of short carbon nanotubes (SCNTs) and reduced graphene flakes (SRGO) encapsulation. Reproduced with permission [68]. Copyright 2021, ACS. (b) Polymer‐derived carbon coating using the sol–gel method. Reproduced with permission [69]. Copyright 2023, ACS. (c) Photoinitiated fabrication of yolk–shell polypyrrole‐iron coated porous Si microspheres. Reproduced with permission [70]. Copyright 2023, Elsevier. (d) PVP‐derived phenolic‐rich carbon interlayer formed through grafting and thermal treatment. Reproduced with permission [71]. Copyright 2024, ACS.

Various surface‐engineering strategies have been explored to overcome the challenges of porous μ‐Si (Figure 11). Direct CNT growth on porous Si by catalytic CVD yielded a low ICE of 60.2% [72], consistent with as‐grown CNTs’ typically low ICE from high surface area [73]. To address this, a weave‐cage carbon network of short CNTs plus reduced graphene flakes (PSi/SCNT‐SRGO) [68] was developed (Figure 11a), which demonstrated improved ICE to 81.1% and retained 1008 mAh g^−1^ after 600 cycles at 1 A g^−1^ (~60% retention). Sol–gel‐derived polymer carbon coatings on porous Si (from acid‐etched Si–Al alloy) (Figure 11b) [69] showed better durability—725 mAh g^−1^ after 300 cycles at 1 A g^−1^ (83.7% retention)—but its average CE of ~99.35% remains below practical targets. In a different strategy, a photoinitiated fabrication approach was used to synthesize yolk–shell polypyrrole‐iron‐coated porous Si microspheres (PSi@PPy‐Fe) (Figure 11c) [70]. The method drives interfacial‐selective pyrrole polymerization on Si, yielding strong adhesion; XPS and DFT indicate hydrogen‐bonding and covalent linkages that enhance charge transfer, Li^+^ transport, and mechanical integrity. The electrode retained 1853.7 mAh g^−1^ after 200 cycles at 1 A g^−1^ (90.7% retention) and maintained 1558.4 mAh g^−1^ at 4 A g^−1^. Explicit reporting of CE is necessary for a more comprehensive evaluation.

Other recent studies have explored alternative strategies, including CNT wrapping followed by carbon coating [74], nitrogen‐doped carbon coatings [75], hierarchical confinement using Ti_3_C_2_T_*x*_ MXene/rGO with an outer CVD carbon coating [76], and, notably, an ultrathin phenolic carbon interlayer coating [71]. In the MXene/rGO/CVD‐carbon design [76], porous μ‐Si derived from Si–Al dealloying retained 992 mAh g^−1^ after 200 cycles at 1 A g^−1^, although the ICE remained relatively low at 63.7%. Notably, Mahesh et al. [71] synthesized porous μ‐Si via metal‐assisted chemical etching (MACE), followed by PVP grafting and thermal treatment under N_2_ to form a phenolic‐rich carbon interlayer (Figure 11d). This yielded a covalently bonded Si·O·C/Si·C interface: Si·C linkages reinforce structural integrity, while Si·O·C sites promote Li^+^ adsorption. As a result, the m‐GPSi anode achieved 3134 mAh g^−1^ and retained 80% capacity after 100 cycles at 0.1 A g^−1^, with an ICE of 90%, while the CE reached ~99% by 10–15 cycles. However, improved cycling capacity retention and longer‐term CE must still be demonstrated for practical viability.

μ‐Si anodes offer clear advantages—higher tap density, lower surface area, and lower cost—and coatings/porous architectures have improved performance; however, reported metrics still fall short of commercial benchmarks. Initial CEs often remain ≤~90%; long‐term CE is rarely sustained at ≥~99.95%; early‐cycle capacity loss from fracture‐induced loss of electrical connectivity is common; and dense μ‐Si is often transport‐limited, which lowers specific capacity. Advances in ICE, long‐term CE, and durability are still needed to realize the full potential of μ‐Si anodes in next‐gen LIBs.

### 4.2 Structured Silicon Anodes

Structured silicon refers to geometry‐engineered architectures—primarily silicon nanowires and thin‐film silicon on 3D scaffolds—designed to accommodate lithiation‐induced volume expansion while preserving electrical continuity and interfacial stability. These architectures leverage size and geometry control: subcritical feature sizes reduce stress accumulation and suppress fracture; nanowires accommodate strain largely through radial expansion, and thin films use conductive scaffolds to maintain electron pathways and provide space for volume expansion during cycling. Compared with conventional particle‐based electrodes, these approaches use geometry and architecture as the main design tools to improve mechanical stability, transport kinetics, and cycling performance.

Multiple commercial efforts now pursue structured silicon (e.g., CVD‐grown nanowires, nanowires integrated onto graphite hosts, and porous Si fiber/3D‐scaffold designs), reflecting a shift from particulate electrodes toward architecture‐first design. Because many industrial results are proprietary and not directly comparable, the discussion that follows draws on peer‐reviewed data, emphasizing practical ICE/CE, cycling stability, areal/tap‐density targets, and post‐calendering integrity. Table 3 summarizes the major practical performance metrics reported for structured Si architectures.

**TABLE 3:** Summary of reported structured silicon anodes, including silicon nanowires and thin‐film silicon architectures on three‐dimensional scaffolds.

References	Structure	Tap density, g cm^−3^	Mass loading, mg cm^−2^	Current density, A g^−1^	Initial capacity, mAh g^−1^	ICE, %	CE, %	Cycles	Capacity retention, %
Silicon nanowires									
[77]	Si@Void@SiO_2_	—	0.8	2.1	1981	74	99.8	200	111
[78]	c‐Si NWs/CF	—	1.2−1.5	1	3127	89	—	500	66
[79]	Nanotextile 100% Si	—	0.8–1.4	0.7	2158	59	99.5	200	76
[80]	Si–C sphere	0.55	—	4.2	~2000	90	—	1000	100
Si thin film									
[81]	CNT–Si core–shell	—	—	0.84	2768	—	—	100	91
[82]	VGs@Si@PCFs	—	1	1	1025	83.8	—	3000	84
[83]	N‐G@Si‐30@HS	—	0.3	2.1	~1500	81	—	1400	86
[84]	CNT@Si@C spheres	0.5	0.53	1.9	~1500	—	99.9	1500	87

#### 4.2.1 Silicon Nanowires (SiNWs)

SiNWs exploit a 1D geometry that shortens Li^+^/electron pathways, distributes stress more effectively, and improves mechanical flexibility—making them attractive high‐performance anodes. As with nanoparticles, surface passivation is essential to limit parasitic reactions and stabilize the SEI, and volume‐change management is required to avoid SEI fracture. Accordingly, SiNWs have been engineered in core–shell and yolk–shell formats.

**FIGURE 12:**
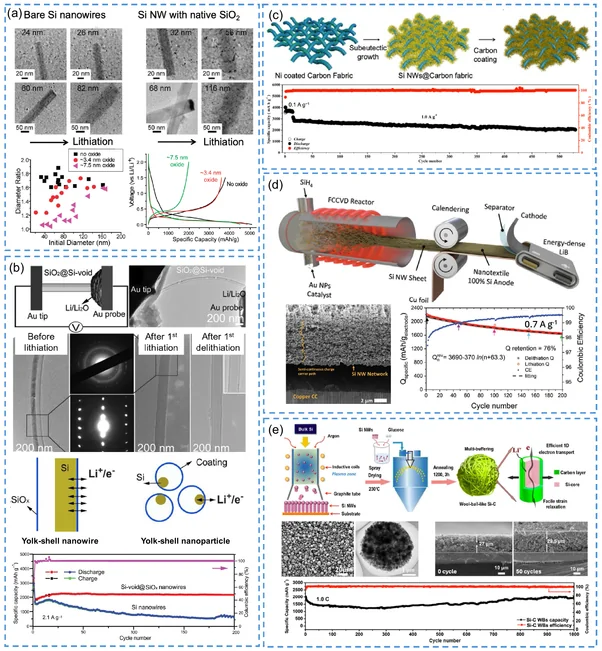
SiNW‐based anodes: (a) Si@SiO_2_ core‐shell. Reproduced with permission [85]. Copyright 2011, ACS. (b) Si@Void@SiO_2_ yolk‐shell. Adapted with permission [77]. Copyright 2017, Wiley. (c) Carbon‐coated SiNWs on carbon fabric as self‐supported electrodes. Adapted with permission [78]. Copyright 2017, ACS. (d) Nanotextile 100% Si anodes from interconnected SiNWs. Reproduced with permission [79]. Copyright 2024, Wiley. and (e) Secondary microspheres assembled from SiNWs. Reproduced with permission [80]. Copyright 2018, ACS.

McDowell et al. [85] evaluated Si@SiO_2_ core–shell nanorod structures (Figure 12a) and revealed that SiO_2_ coating can suppress volume expansion to some extent, as shown by TEM imaging of nanorods before and after lithiation. A ~3.4 nm SiO_2_ shell suppressed expansion without sacrificing capacity; thicker, insulating shells impeded charge transport and reduced capacity. For larger‐diameter rods, SiO_2_ was less effective at strain control. Likewise, photolithographically structured SiNWs [86] (diameter ~630 nm) showed poor cycling stability even with a carbon coating, underscoring the diameter dependence of stability.

To better accommodate expansion, yolk‐shell nanowire structures have been explored. Qian et al. [77] first synthesized Si–ZnS@SiOx nanowires via thermal evaporation, and then selectively etched ZnS with hydrochloric acid to create voids, yielding a Si–void@SiOx structure. The mechanically robust SiO_*x*_ shell buffered volume change, and, unlike yolk–shell nanoparticles that often suffer point contact, the line contact along the wire enabled efficient ion/electron transport as shown in Figure 12b. As a result, the electrode delivered 2197 mAh g^−1^ after 200 cycles at 2.1 A g^−1^ with no capacity decay reported, demonstrating excellent cycling stability. Despite a low ICE of 74%, an impressive CE of nearly 99.8% was achieved after 25 cycles. Applying a thin, conformal passivation layer that isolates the native SiO_2_ from the electrolyte can raise ICE by suppressing SEI formation. Similarly, a NW@C yolk–shell variant [87] has also been explored, but its cycling stability was insufficient, likely because a centered wire detaches from the shell as observed in TEM imaging, limiting contact and transport.

SiNWs have also been explored for self‐supported electrodes. For example, carbon‐coated SiNWs grown on carbon fabric (Figure 12c) [78] formed a binder‐free, flexible electrode with  ~3200 mAh g^−1^ at 0.5 A g^−1^ and ~66% retention (2021 mAh g^−1^) after 500 cycles at 1.0 A g^−1^. Because the reported specific capacity excludes the mass of the carbon fabric, the actual energy density is lower. This design could be viable for low‐capacity/flexible batteries, but without explicit CE data, a full performance evaluation remains uncertain. Another self‐supported electrode, a 100% Si “nanotextile” synthesized via floating catalyst chemical vapor deposition (Figure 12d) [79], reached ~2150 mAh g^−1^ at 0.7 A g^−1^, retaining 76% capacity after 200 cycles, while CE steadily rose beyond 99.5%. Nevertheless, its low ICE of only 59% indicates substantial irreversible Li consumption arising from direct Si exposure, necessitating passivation strategies to improve ICE and long‐term cycling performance.

A morphology resembling the secondary microsphere has also been explored. Guolin et al. [80] assembled wool‐ball‐like SiNW–C microspheres (Figure 12e) by RF‐plasma growth of SiNWs followed by spray‐drying with glucose and calcination, yielding hierarchical Si–C spheres with tap density of 0.55 g cm^−3^. The electrodes sustained ~2000 mAh g^−1^ after 1000 cycles at 1 C (no reported fade) with ~90% ICE. In situ TEM measured ~19.5% volume change; mechanical tests indicated internal voids provided elasticity and preserved connectivity—features compatible with calendering. The cycling plot shows an initial capacity drop followed by a gradual increase, which may not occur in a practical full cell due to limited lithium inventory. Since CE is not explicitly reported, its long‐term viability remains uncertain, but its low surface area suggests that the CE could be high, as seen for SiNPs secondary microspheres.

SiNWs can combine high‐rate capability with mechanical resilience when diameter, contact geometry, and shell thickness are tuned. SiNW secondary microspheres formed from short wires (high tap density) align well with existing slurry‐cast/calendering lines. Further progress will depend on an optimized passivation coating to lift ICE/CE and suppress early fade, and on integrating engineered NW architectures (e.g., yolk–shell) into secondary microspheres to maintain contact and extend cycle life.

#### 4.2.2 Si Thin Films on 3D Scaffolds

Thin‐film Si has long been explored as a promising alternative to particulate Si. Early CVD work (1999) [88] demonstrated that a micron‐thick a‐Si is a viable anode, but capacity faded rapidly—from ~1000 to ~200 mAh g^−1^ in 20 cycles. Interest grew after Graetz et al. [89] reported markedly better stability for ~100 nm evaporated a‐Si (~3500 mAh g^−1^ initially, ~2000 mAh g^−1^ after 50 cycles), underscoring the benefits of sub‐micrometer thickness and interface control. The trade‐off, however, is low areal capacity for the thinnest, most stable films—motivating the shift to 3D scaffolded architectures to increase active loading and achieve practical areal capacity (mAh cm^−2^).

**FIGURE 13:**
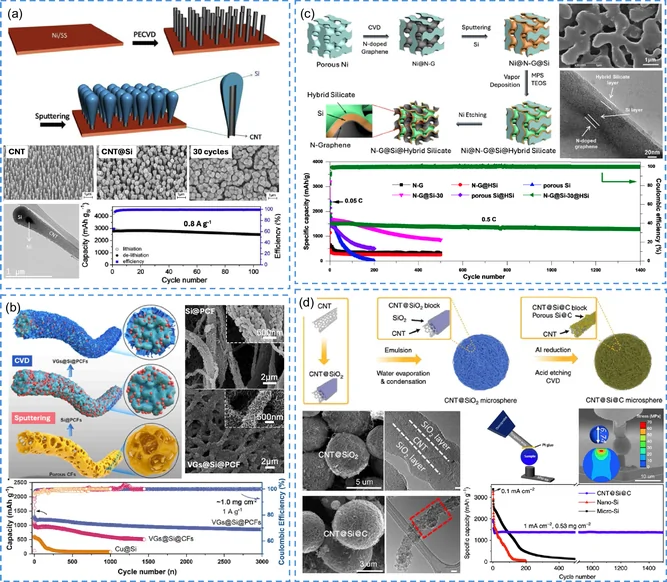
Si thin‐film‐based 3D anodes: (a) Si coating on vertically aligned CNTs. Reproduced with permission [81]. Copyright 2013, Elsevier. (b) Si on carbon nanofibers protected by nitrogen‐doped vertical graphene nanosheets. Reproduced with permission [82]. Copyright 2023, Elsevier. (c) Sandwich‐structured N‐doped graphene@Si@hybrid silicate anode with a bicontinuous porous nanoarchitecture. Reproduced with permission [83]. Copyright 2020, ACS. (d) Secondary microspheres: hierarchical CNT@Si@C spheres [84]. Copyright 2020, Springer Nature.

For example, Fan et al. [81] sputtered Si onto vertically aligned CNTs, forming a vertically opened pore structure that enhanced strain accommodation and Li^+^ transport (Figure 13a). This design exhibited an initial capacity of 2768 mAh g^−1^ at 0.84 A g^−1^, retaining 2502 mAh g^−1^ after 100 cycles (90.4% retention), indicating excellent cycling stability. Additionally, the electrode maintained 1500 mAh g^−1^ even at 16.8 A g^−1^, demonstrating excellent rate capability. The high surface area of CNTs may have added capacity via the electric double‐layer capacitance mechanism [90]. While the cone‐shaped Si coating improved structural stability, microcracking was observed after only 30 cycles due to nanorod crowding, as shown in Figure 13a, highlighting the need for controlled porosity. Given the high surface area of Si/CNT architectures, extensive SEI formation can consume lithium, underscoring the need for thin, protective passivation layers. Extending this idea, sputtered Si on porous CNFs bridged by N‐doped vertical graphene nanosheets (VGs@Si@PCFs) was developed (Figure 13b) [82]. The electrode delivered 2205 mAh g^−1^ at 0.1 A g^−1^ and 83.5% retention (856.7 mAh g^−1^) after 3000 cycles at 1.0 A g^−1^ (CE not reported explicitly). To further leverage sputtered Si coatings, a sandwich‐structured N‐doped graphene@Si@hybrid silicate porous nanoarchitecture was developed (Figure 13c) [83]. N‐doped graphene (N‐G) was first grown by CVD on a nanoporous Ni template. Si was then sputtered onto the N‐G, followed by vapor‐phase deposition of a hybrid silicate layer. Finally, the Ni template was etched away to obtain a free‐standing N‐G@Si@HSi film. The anode delivered ~1286 mAh g^−1^ and operated for >1400 cycles without significant fade. The ICE was 81.0%, and CE exceeded 99% after ~10 cycles, though precise CE reporting is needed for practical assessment.

To raise packing density and withstand calendering, Si‐coated nanocarbons have been assembled into secondary microspheres. CNT@Si@C spheres (Figure 13d) were made by SiO_2_ sol–gel coating on CNTs, emulsion assembly, aluminothermic reduction to Si, and a final CVD carbon shell (tap density ~0.50 g cm^−3^) [84]. The microsphere‐based electrode delivered ~1500 mAh g^−1^ and retained 87% over 1500 cycles at 1 mA cm^−2^. The spheres expanded by only ~40% upon lithiation and withstood >200 MPa compressive load without fracture—performance compatible with industrial calendering and indicative of practical viability for LIBs.

Thin‐film Si on structured hosts can pair high specific capacity (≈1500–2500 mAh g^−1^) with long life (>1000 cycles). Moving toward practical deployment will require a surface passivation strategy to reduce first‐cycle loss and sustain high long‐term CE on high‐surface‐area scaffolds, together with densification where porosity limits volumetric capacity. As a more drop‐in route for traditional graphite‐anode manufacturing, these materials can be assembled into secondary microspheres to achieve high tap/electrode density and calendering robustness.

## 5 Summary and Perspective

Our critical evaluation of the literature indicates that Si‐dominant anodes have made substantial progress toward practical implementation. Across nanoparticles, micro‐silicon, and structured silicon architectures, several approaches now demonstrate promising combinations of capacity, cycling stability, and electrode‐level performance. In particular, secondary microsphere architectures assembled from nanoparticles or structured‐Si building blocks appear especially attractive because they simultaneously reduce electrolyte‐exposed surface area, improve tap/electrode density, preserve short Li‐diffusion lengths, and remain largely compatible with existing slurry‐casting and roll‐to‐roll manufacturing processes. From a manufacturability perspective, such drop‐in architectures may offer a more practical route to commercialization than highly specialized electrode designs requiring entirely new manufacturing infrastructure.

Nevertheless, translating encouraging half‐cell results into commercially viable full cells remains the central challenge. Although several studies have demonstrated full‐cell performance in coin‐cells exceeding ~500 Wh kg^−1^ based on electrode‐level mass accounting, such values do not necessarily represent practical packaged‐cell energy density. In real cells, inactive components—including current collectors, separators, electrolyte, tabs, and packaging—contribute substantial mass and reduce the achievable cell‐level energy density. Thus, demonstrating >500 Wh kg^−1^ at the electrode level is an important milestone, but is not by itself sufficient to realize a practical 500 Wh kg^−1^ battery. Achieving this target will require further optimization of both anode and cathode systems, along with careful control of areal capacity, electrode density, swelling, N/P ratio, and inactive‐material fraction.

Long‐term cycle life remains an even greater challenge. Although many architectures achieve high capacities and acceptable half‐cell cycling, sustaining ≥1000 cycles with high‐capacity retention requires exceptionally high CE. Further improvements in binder systems, electrolyte formulations, passivation strategies, and contact‐preserving electrode architectures will be needed to minimize irreversible lithium loss and maintain electrical connectivity throughout cycling. In addition, many of the most successful full‐cell demonstrations rely on prelithiation, highlighting the need for scalable and cost‐effective prelithiation strategies suitable for industrial implementation. Finally, future studies should increasingly emphasize validation in commercially relevant cell formats and loading conditions, since performance demonstrated in coin cells may not directly translate to pouch or larger‐format cells. If these materials‐, electrode‐, and cell‐level challenges can be addressed simultaneously, Si‐dominant anodes offer a credible pathway toward LIBs approaching ~500 Wh kg^−1^ and ≥1000 cycles.

## Conflicts of Interest

The authors declare no conflicts of interest.

## Data Availability

The data that support the findings of this study are available from the corresponding author upon reasonable request.
